# Standardisation of labial salivary gland histopathology in clinical trials in primary Sjögren's syndrome

**DOI:** 10.1136/annrheumdis-2016-210448

**Published:** 2017-06-09

**Authors:** Benjamin A Fisher, Roland Jonsson, Troy Daniels, Michele Bombardieri, Rachel M Brown, Peter Morgan, Stefano Bombardieri, Wan-Fai Ng, Athanasios G Tzioufas, Claudio Vitali, Pepe Shirlaw, Erlin Haacke, Sebastian Costa, Hendrika Bootsma, Valerie Devauchelle-Pensec, Timothy R Radstake, Xavier Mariette, Andrea Richards, Rebecca Stack, Simon J Bowman, Francesca Barone

**Affiliations:** 1 Rheumatology Research Group and Arthritis Research UK Rheumatoid Arthritis Pathogenesis Centre of Excellence (RACE), University of Birmingham, Birmingham, UK; 2 Department of Rheumatology, University Hospitals Birmingham NHS Trust, Birmingham, UK; 3 Broegelmann Research Laboratory, Department of Clinical Science, University of Bergen, Bergen, Norway; 4 Department of Rheumatology, Haukeland University Hospital, Bergen, Norway; 5 Department of Orofacial Sciences, University of California San Francisco, San Francisco California, USA; 6 Centre for Experimental Medicine and Rheumatology, Queen Mary University of London, London, UK; 7 Department of Pathology, University Hospitals Birmingham NHS Trust, Birmingham, UK; 8 Department of Pathology, King's College London, London, UK; 9 Rheumatology Unit, University of Pisa, Pisa, Italy; 10 Musculoskeletal Research Group and NIHR Biomedical Research Centre in Ageing and Chronic Diseases, Newcastle University, Newcastle, UK; 11 Department of Pathophysiology, University of Athens, Athens, Greece; 12 Section of Rheumatology, Casa di Cura di Lecco, Lecco, Italy; 13 Department of Oral Medicine, King's College London, London, UK; 14 Department of Pathology, University of Groningen, Groningen, The Netherlands; 15 Department of Pathology, Brest University Hospital, Brest, France; 16 Department of Rheumatology and Clinical Immunology, University of Groningen, Groningen, The Netherlands; 17 Rheumatology Department, Cavale Blanche Hospital and Brest Occidentale University, ER129, Brest, France; 18 Department of Rheumatology and Clinical Immunology, University Medical Centre Utrecht, Utrecht, The Netherlands; 19 Rheumatology Department, Université Paris-Sud, Assistance Publique—Hôpitaux de Paris, INSERM U1184, Le Kremlin-Bicêtre, France; 20 Department of Oral Medicine, Dental Hospital, Birmingham, UK

**Keywords:** Sjøgren's Syndrome, Autoimmunity, Outcomes research

## Abstract

Labial salivary gland (LSG) biopsy is used in the classification of primary Sjögren's syndrome (PSS) and in patient stratification in clinical trials. It may also function as a biomarker. The acquisition of tissue and histological interpretation is variable and needs to be standardised for use in clinical trials. A modified European League Against Rheumatism consensus guideline development strategy was used. The steering committee of the ad hoc working group identified key outstanding points of variability in LSG acquisition and analysis. A 2-day workshop was held to develop consensus where possible and identify points where further discussion/data was needed. These points were reviewed by a subgroup of experts on PSS histopathology and then circulated via an online survey to 50 stakeholder experts consisting of rheumatologists, histopathologists and oral medicine specialists, to assess level of agreement (0–10 scale) and comments. Criteria for agreement were a mean score ≥6/10 and 75% of respondents scoring ≥6/10. Thirty-nine (78%) experts responded and 16 points met criteria for agreement. These points are focused on tissue requirements, identification of the characteristic focal lymphocytic sialadenitis, calculation of the focus score, identification of germinal centres, assessment of the area of leucocyte infiltration, reporting standards and use of prestudy samples for clinical trials. We provide standardised consensus guidance for the use of labial salivary gland histopathology in the classification of PSS and in clinical trials and identify areas where further research is required to achieve evidence-based consensus.

## Introduction

Labial salivary gland (LSG) biopsy is widely used in the diagnosis of primary Sjögren's syndrome (PSS) and plays an integral role in the established American-European Consensus Group classification criteria^1^ and the proposed American College of Rheumatology/European League Against Rheumatism (ACR/EULAR) criteria.^2^ Evidence suggests that it has the potential to stratify patients,^3–6^ and may have potential as a biomarker in clinical trials.^7^

The most characteristic feature of PSS on biopsy with a sensitivity and specificity of >80%^8^ is focal lymphocytic sialadenitis (FLS), which describes the presence of dense aggregates (foci) of ≥50 mononuclear cells (mostly lymphocytes), in a periductal or perivascular localisation.^9^ A modification based on focus score (FS) calculation was proposed in 1974,^10^ and further work established FS ≥1 (ie, ≥1 focus per 4 mm^2^) for use in classification criteria.^8^ ^11–14^ The Sjögren's International Clinical Collaborative Alliance (SICCA) have published a widely used protocol for sample preparation and the assessment of FS in suspected Sjögren's syndrome .^14^ ^15^ Nevertheless, the initial determination of FLS, prior to calculation of a FS, is still a cause of variability of practice in the SS community.^7^ We previously discussed additional areas of variability including the acquisition and processing of the salivary gland tissue and the histological interpretation of the local infiltrate.^7^ ^16–18^ The SICCA protocol specifies that foci in FLS occur adjacent to normal appearing acini, but features of non-specific chronic sialadenitis (NSCS) such as atrophy and duct dilation are common in the population and so may coexist with PSS. NSCS may also be associated with infiltration of lymphocytes and even aggregation, thus raising issues for interpretation and FS calculation.^7^ ^16^ The SICCA protocol provides no additional guidance beyond FS calculation on the reporting of size of foci, their degree of organisation, germinal centres (GCs) and lymphoepithelial lesions (LESA), the latter being characterised by lymphocytic infiltration of ducts and basal cell hyperplasia resulting in a multilayered epithelium, and which may also have prognostic significance.^19^

The goal of this study was to develop a process of standardisation in order to confirm areas of consensus and highlight areas of uncertainty, with a view to stimulating further research and evidence-based recommendations. A few centres use parotid gland biopsies^6^ but we have focused this work on LSG tissue, as this remains the most commonly employed in clinical practice.^20^ In this study, we do not address the biopsy procedure itself but focus on the processing of the tissue and measurement of PSS-related inflammation. The target users include histopathologists, rheumatologists, oral medicine and oral and maxillofacial surgeons and ophthalmologists, as well as pharmaceutical companies planning clinical trials in PSS.

## Methods

The standard operating procedures produced by EULAR on guidelines development in rheumatic and musculoskeletal disorders broadly formed the basis of the process followed.^21^

### Item development

A literature review was carried out by the steering committee members of the ad hoc working group. This has been published and identified points of outstanding uncertainty for discussion.^7^ These comprised the agenda items for the workshop.

### Workshop

A 2-day workshop was held in February 2014 in Birmingham, UK.

#### Day 1

The first day focused on histopathology in the diagnosis of PSS. Presentations addressed the rationale for biopsy, histological features and scoring systems, challenges and variability in application of the SICCA protocol, GCs and LESA, and methods for measuring change in biopsies in clinical trials as a prelude for day 2. There were 23 clinical expert attendees with a background in rheumatology, histopathology or oral medicine. The relevant issues were interactively discussed to establish a draft framework of points to consider in the areas of (1) glandular tissue requirements, (2) criteria defining FLS and FS and (3) assessment of GCs and LESAs.

#### Day 2

A larger number of attendees were present on the second day (n=39), representing an increased number of specialists with an interest in PSS clinical trials and including a clinical trials statistician, a health psychologist and three patient partners. Presentations summarised issues around the scoring of biopsies, additional pathological features that may be relevant to clinical trials and the natural history and reliability of histopathological changes. Breakout groups and roundtable discussion were used to propose points relevant to clinical trials in the areas of (1) calculation of focus size, (2) additional parameters that could be measured, (3) reporting standards and (4) timing of biopsy and requirement for a placebo group. In a parallel session facilitated by a health psychologist, the patient partners discussed the acceptability of LSG biopsies as a clinical trials outcome measure. A concluding discussion addressed the agenda for future work.

### Delphi process

The provisional points gathered at the workshop were substituted for a traditional first round of a Delphi process.^22^ These were reviewed and edited for clarity and completeness by a subgroup of six experts. A subsequent eDelphi round was conducted with 50 experts (comprising the original workshop attendees together with additional experts). These were asked to rate 20 points on a 0–10 scale, where 0 indicated no agreement and 10 complete agreement, and to provide explanation when there was disagreement. Points were divided into those of general application, and those most relevant to a clinical trials setting. Explanatory text and selected references accompanied the points.

### Analysis

All the points were graded, based on available evidence, according to the scale (A–D) recommended by the Oxford Centre for Evidence-based Medicine.^23^ The available evidence has been previously reviewed.^7^ Agreement was defined as a mean score of ≥6 and ≥75% of respondents scoring ≥6.

## Results

**Figure 1:**
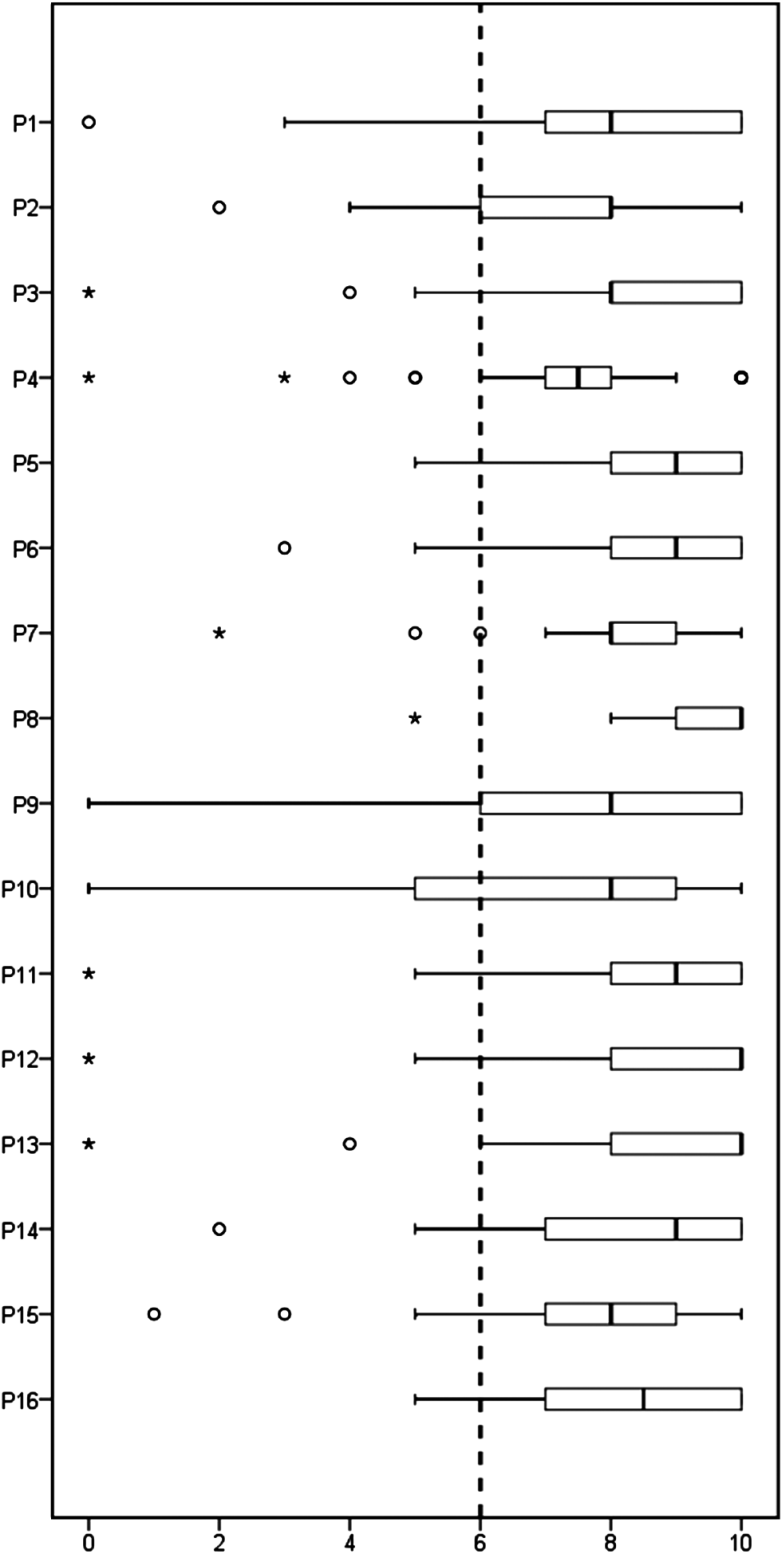
Box plot of the 16 agreed points (table 1) on the vertical axis and level of agreement (0–10) on the horizontal axis. The dashed line shows the predefined cut-off for agreement. Boxes indicate first and third quartiles with the internal line indicating the median. Whiskers indicate the minimum and maximum scores given except when considered outliers, whereas circles indicate outliers (≤1st quartile–1.5×IQR) and stars far outliers (≤1st quartile–3×IQR).

A total of 39 experts (78%) responded to the eDelphi exercise, of which 22 identified their principal specialty as rheumatology/internal medicine (54%), 11 as oral medicine (30%) and 6 (16%) as histopathology. Nine rheumatologists (41%) and four (36%) oral medicine specialists described their experience of actually reviewing LSG histopathology as moderate, 8 (36%) and 2 (18%) as extensive. No discrepancy in responses was noted between specialty groups. Following the eDelphi exercise, a total of 16 points (table 1) met the criteria for agreement. These are listed in table 1, together with the strength of recommendations, and expert scores. The spread of scores is illustrated in figure 1. These points are discussed below. Points not meeting criteria for agreement are listed in online supplementary table S1.

10.1136/annrheumdis-2016-210448.supp1 supplementary table

**Table 1:** Consensus guidance divided into points of general application and those more relevant to clinical trials, showing strength of recommendation (A–D) based on available evidence, according to the scale (A–D) recommended by the Oxford Centre for Evidence-based Medicine^23^

Point		Strength of recommendation	Number of respondents	Mean score (SD)	% ≥6
General guidance					
1	The minimum number of minor salivary glands is suggested to be four (six if small), and should be surgically separated	D	39	8.0 (2.4)	82
2	The minimum surface area of gland sections examined should be 8 mm^2^	D	39	7.5 (1.9)	82
3	If the first cutting level is inconclusive, or in the context of a clinical trial, consideration should be given to including two additional cutting levels at 200 µm intervals (typical focus diameter is <50 μm) in order to increase the surface area	C/D	37	8.2 (2.0)	92
4	Care should be given to preparation of paraffin blocks, with smaller glands set higher to allow midspecimen sampling during cutting	D	38	7.5 (2.1)	87
5	Histological examination should determine whether there is FLS present. Attribution of FLS, or possible FLS, should be followed by calculation of a focus score	B	39	8.8 (1.4)	95
6	The extent (absent, mild, moderate, severe) of atrophy, fibrosis, duct dilatation and non-specific chronic sialadenitis, in addition to the presence or absence of FLS, should be reported	C	39	8.5 (1.7)	92
7	Calculation of the focus score should include the whole of the glandular surface area in the denominator, to avoid introduction of bias	D	39	8.3 (1.6)	95
8	The presence or absence of germinal centre-like structures and lymphoepithelial lesions should be reported	C	39	9.5 (1.0)	97
Guidance relevant to clinical trials					
9	The Focus score should be recorded, and the area of individual foci should also be summed and divided by glandular area to give a more quantitative indication of the extent of glandular infiltration	C	38	7.5 (2.5)	76
10	Once FLS has been confirmed, all foci should be included in the Focus score and in area of foci calculations, even when adjacent to abnormal acini or ducts, to avoid introduction of bias	D	38	7.3 (2.6)	76
11	Staining for CD3, CD20 and CD21 should be included, and the presence of germinal centre-like structures should be reported as the proportion of foci with both T/B-cell segregation and follicular dendritic cell networks. Consideration should be given to reporting the mean B/T cell ratio in foci	C/D	38	8.1 (2.0)	89
12	Scoring should be undertaken by two trained observers who have reviewed a reference slide set, and with reporting of intraobserver and interobserver variability	D	38	8.9 (1.9)	95
13	Samples should be scored blind to subject and order	D	36	8.8 (2.1)	94
14	High-resolution image morphometry of each sample should be stored	D	38	8.2 (2.0)	89
15	Given the stable or slowly progressive nature of the histological features, baseline biopsies may be substituted with prior biopsies to reduce the number of biopsies required. However, given the limited evidence available, these should have been acquired no longer than 1 year prior to baseline	C	38	7.8 (2.0)	87
16	The optimal period of time for rebiopsy has not been established and will depend on the agent employed.	D	39	8.3 (1.6)	92

The level of agreement (0–10 scale) among participants is also shown, represented by mean scores and the percentage of respondents who scored the point ≥6/10.

FLS, focal lymphocytic sialadenitis.

### Glandular tissue requirements

Given the scattered nature of foci, it is important that there is sufficient material available to allow a robust and reliable analysis. In point 1 (table 1), we propose obtaining a minimum of four LSGs, unless these are small (<2 mm), in which case six glands should be obtained if feasible. Three respondents argued for the use of fewer glands (two to three), and two for a greater number (five to seven).

A minimum glandular surface area to be examined of 8 mm^2^ was proposed to facilitate agreement. This minimum should comprise good quality glandular tissue. In the case of an inconclusive biopsy, for example, uncertainty over FLS, borderline FS for diagnosis or insufficient surface area, then two additional cutting levels could be employed (point 3). Glandular surface area from a single cutting level of multiple glands may also be optimised by aligning glands during preparation of the paraffin blocks (point 4).

### Assessment of FLS and FS

**Figure 2:**
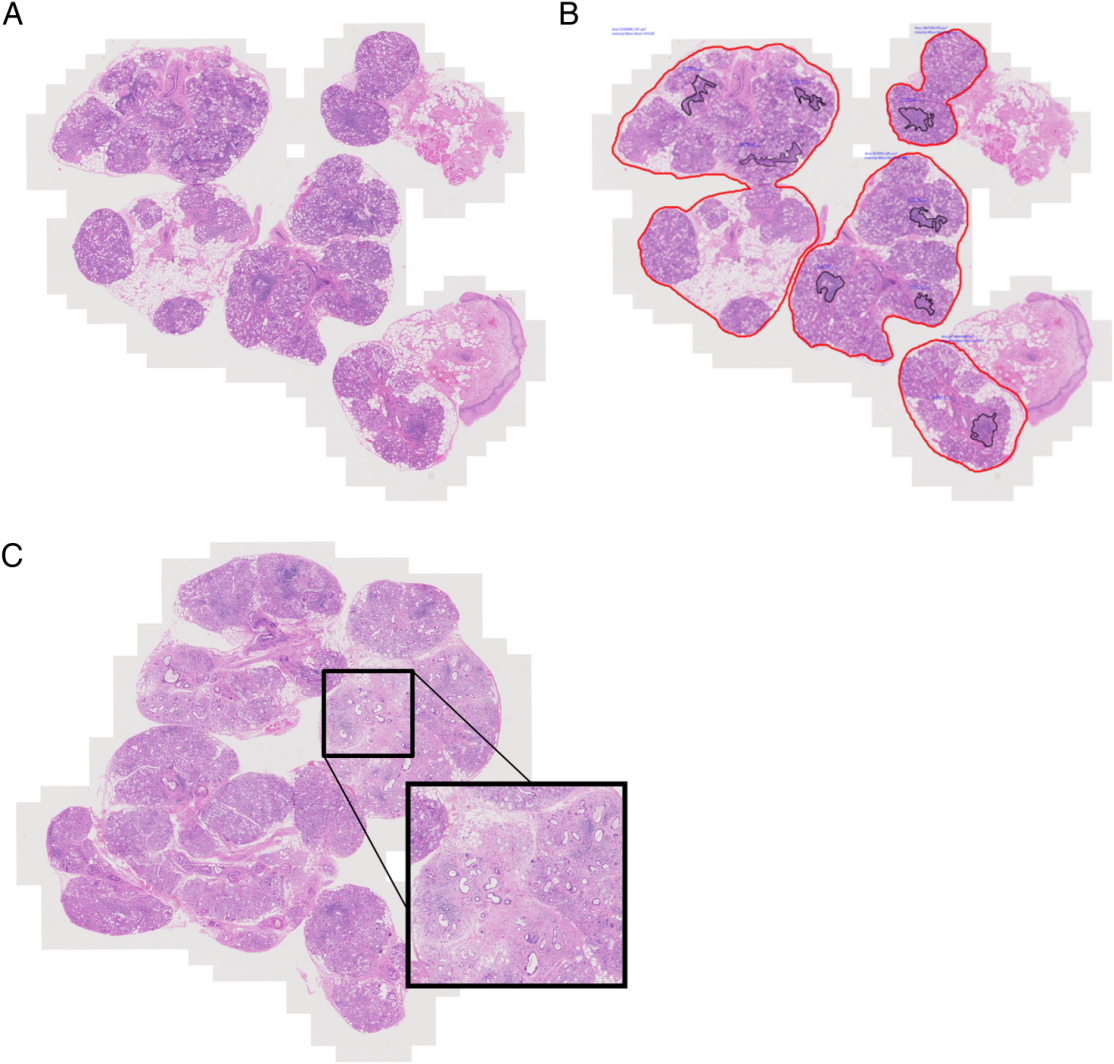
(A) Microphotograph illustrating salivary gland biopsy obtained from a patient with primary Sjögren's syndrome, stained with H&E. (B) Image analysis applied to macrosection showing delineation of glandular tissue in *red*. Focus score is calculated by counting the number of foci, whose area is delineated within the *black lines*, dividing by the whole glandular surface area in mm^2^ and multiplying by 4 to give the number of foci per 4 mm^2^ over the whole glandular area. In this example, the glandular surface delineated includes interspersed atrophic areas but excludes any attached epithelial or connective tissue. The measured glandular area is 20.89 mm^2^ and there are 8 foci giving a focus score of 1.53. (C) Microphotograph illustrating salivary gland biopsy obtained from a patient with diagnosis of primary Sjögren's syndrome that presents a large area of fibrosis and parenchymal atrophy, alongside areas of focal lymphocytic sialadenitis (original magnification ×20).

The presence of FLS should be determined prior to FS calculation (point 5) (figure 2).^14^ Foci may be confluent and foci of any size may include plasma cells, although there was some divergence of expert opinion regarding the extent of plasma cell infiltration that is compatible with FLS. FLS cannot be attributed when the histological appearance of the glands is dominated by features associated with NSCS, such as acinar atrophy, duct dilation and fibrosis, with no evidence of any foci being adjacent to normal parenchyma. Conversely, given the prevalence of NSCS, some foci in PSS may be expected to be adjacent to atrophic features. Expert recommendation is that the extent of the atrophic features should be graded and reported to aid the referring clinician in their interpretation (point 6).

In order to calculate the FS, the total number of foci in the specimen is divided by the glandular surface area, and multiplied by 4, to give the number of foci per 4 mm^2^ (figure 2) Above a FS of 10, foci are typically confluent, and a ‘ceiling’ score of 12 may be applied. Glandular area can be measured with a calibrated eyepiece grid,^15^ but measurement-validated microscope-associated software may also be employed (figure 2B). An important decision is whether to include apparent foci in areas of atrophy, duct dilation and fibrosis, and whether to include the background area in the glandular surface area denominator. Different approaches include excluding infiltrate in compact fibrosis but including that around abnormal acini and ducts, excluding all foci and abnormal areas from the numerator and denominator, or including all. This decision will have an impact on the calculated FS.^16^ There was agreement that FS calculation should include the whole of the glandular surface area in the denominator, including abnormal areas, to avoid introduction of bias (point 7; figure 2B). This includes areas of fibrosis, which cannot reliably be removed from the glandular surface area denominator, although their inclusion may have the potential to reduce the FS, meaning that some patients with a FS ≥1 may become <1 at a late stage of disease (figure 2C).^24^ There was also agreement that in the case of PSS clinical trials at least, the least biased approach and the one likely to have the greatest reproducibility, would be to assume all foci are PSS-related and to be included in the FS (point 10). In the case of repeated biopsies, the patients themselves serve as an internal control. Arguably, this could also be applied to clinical diagnosis, once the presence of FLS has been determined.

### Calculation of focus area

There was support for using the area of mononuclear cell infiltration in addition to the FS as a biomarker in clinical trials (point 9). Data can be presented as percentage of total area infiltrated and mean focus size. This could be achieved with digital analysis of H&E or CD45 immunostaining.

### Ectopic GCs and LESAs

There was strong agreement that the presence of GCs should be reported in routine practice (point 8). However, some respondents commented on the need for a clear definition of these structures. H&E is considered sufficient to allow accurate detection of a fully formed GC by a histopathologist, although additional staining can be used such as B-cell lymphoma 6 (BCL-6) and CD21.

**Figure 3:**
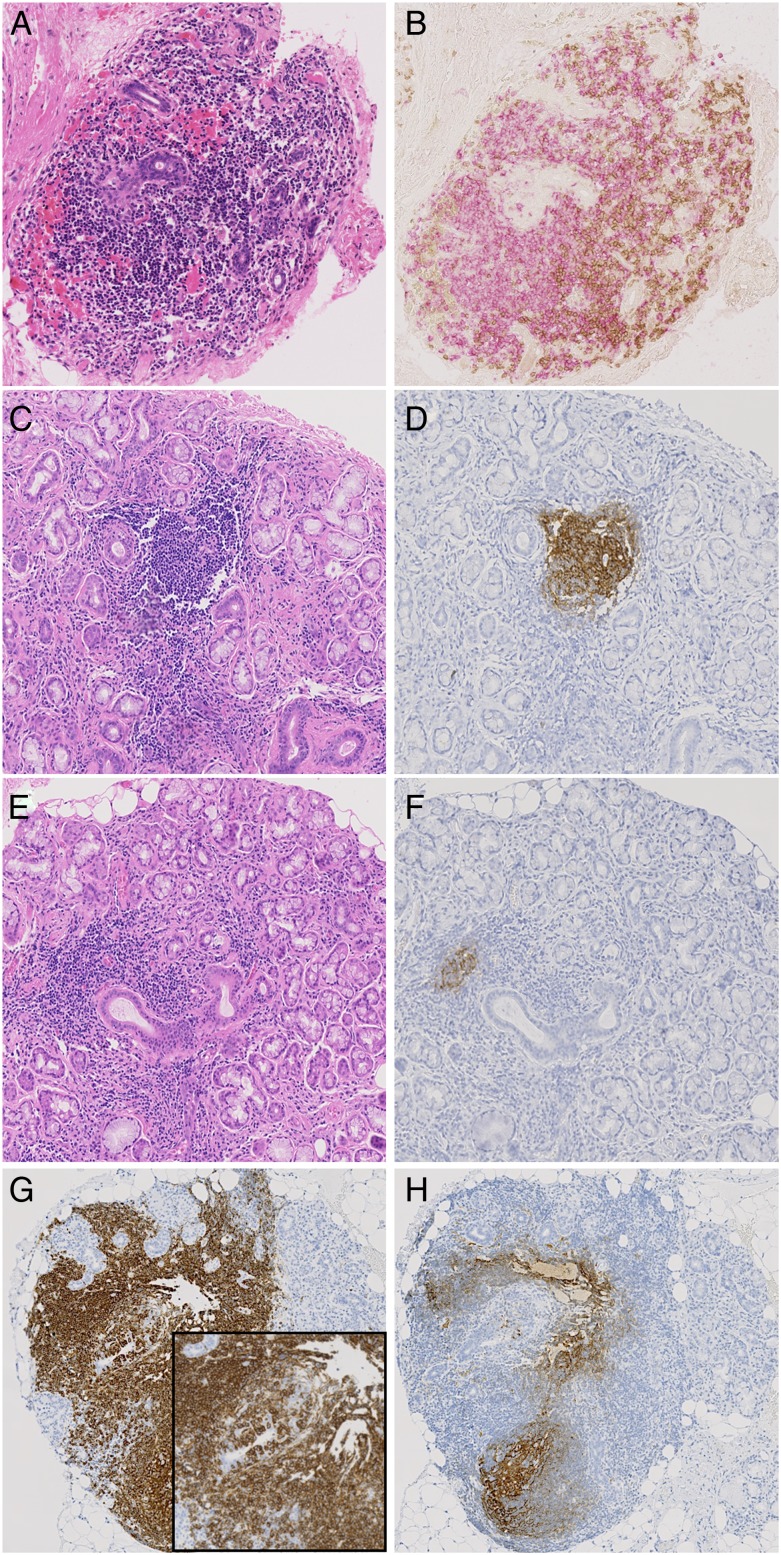
(A–H) Sequential sections illustrating inflammatory infiltrates in the salivary glands of patients with primary Sjögren's syndrome stained by H&E (A, C, E), CD3 (*brown* in B), CD20 (pink in B and *brown* in G) and CD21 (*brown* in D, F and H). (A and B) Sequential section illustrating segregation in T and B cells in large periductal infiltrate in absence of germinal centre (GC). (C and D) Evident GC in H&E stained section confirmed by CD21 staining on sequential section. (E and F) Small CD21+ cluster of follicular dendritic cells (FDCs) in sequential section of a large aggregate with absence of obvious GC features at the H&E staining. (G and H) Large CD20+ infiltrate with obvious lymphoepithelial lesions (inset) and the presence of CD21+ FDC networks at the sequential section.

In the context of clinical trials, we have suggested additional staining with CD21, a marker of follicular dendritic cells (FDCs) and CD3 and CD20 to better define the presence of GCs (point 11) (figure 3). While CD21 long isoform staining by itself does not indicate the presence of a GC, the presence of a FDC network together with B-cell and T-cell segregation would be expected in all,^25^ and this combined approach would avoid the recently highlighted risk of overestimating GCs if relying on CD21 alone.^26^ ^27^

Staining for CD3 and CD20 will also allow calculation of the B/T cell ratio in foci (point 11). While this alone would be insufficient to indicate the degree of segregation, it can be readily measured with digital recognition software.

### Additional parameters for clinical trials

Although some workshop participants were strong advocates of measuring the proportion of IgA and IgG plasma cells, this did not receive sufficient support (mean 6.6; 65% ≥6). Some evidence suggests that the IgA:IgG plasma cell ratio may have diagnostic utility, based on the assumption that IgA plasma cells are normal within the gland (producing secretory IgA) with the role of IgG plasma cells being unclear,^28–31^ but arising as a consequence of chronic inflammation.^32^ However, more background work is required to understand its diagnostic utility and biomarker potential.

Glandular epithelial cell human leukocyte antigen class II expression appears directly related to local T-cell activation and interferon-γ production, and might therefore function as a biomarker.^33^ ^34^ However, again there was insufficient support for measuring this routinely (mean 6.3; 65% ≥6).

### Reporting standards for clinical trials

We recommend that clinical trials using the FS have two observers who report their interobserver variability (point 12) and who score samples blind to subject and chronological order (point 13). Ideally, high-resolution image morphometry of each sample should be stored to facilitate future comparative studies (point 14).

### Timing of biopsy and placebo groups in clinical trials

It was agreed that pre-existing diagnostic biopsies could be substituted for baseline biopsies, provided that sufficient material of acceptable quality was available (point 15). Remarkably, little data exist on the natural history of histopathological changes in PSS.^7^ Therefore, the 1-year cut-off proposed is arbitrary. An optimal period for rebiopsy has not been determined and may depend on the agent studied (point 16). A 6-month timeframe seems reasonable with 3 months being a minimum.

Despite this apparent stability, little is known about variation in scores with repeat sampling, and so it was proposed to retain placebo groups even when histology was the primary outcome, until further experience with heterogeneity of sampling was available. Overall, there was strong support for this, with scores close to the defined agreement (mean 6.8 and 74% rating ≥6). However, a number of responses were strongly negative. Ethical concerns were raised about performing repeat biopsies on patients treated with placebo. Furthermore, it was argued that even in the absence of a placebo group, an improvement in biopsy scores in a small early phase study would still provide a positive go/no-go decision and justify further work.

### Patient perspective on biopsies in clinical trials

Our patient partners felt that two biopsies over a 12-week period would be acceptable, although the rationale and objectives should be clearly explained and feedback of the results would be valued. The ability to use pre-existing samples where available was considered important.

### Agenda for future work

- Development of a web-based reference slide set.
- Establish variability of assessments over short time frames using placebo arms of clinical trials. Using these data determine a minimum number of subjects and minimum detectable difference.
- Establish optimal glandular surface area requirements/number of LSGs.
- Multicentre study of interobserver variability
- Further comparative work between parotid and LSG biopsies.
- Agreement on immunohistochemistry staining protocols, particularly regarding identification of GCs.
- Future application of these assessments in ‘positive’ clinical trials is required to establish discriminant validity.
- Revision of this guidance will be undertaken when significant new data are available from the above studies.
- Further work on the natural history of FLS in PSS and the potential biomarker role of IgA, IgG and IgM plasma cells would be desirable.
- Importantly, the patient perspective will be further studied and morbidity data collected.

## Discussion

Standardising histopathological assessment in PSS is an important objective for routine diagnosis and for clinical trials, to ensure homogeneity of study populations, and improve reliability of assessments and comparability between studies. Extensive work on this topic has been performed by the members of the SICCA group.^14^

Despite so many years of use, however, there remains considerable lack of data regarding the natural history of the histopathological changes associated with PSS, the test reliability or repeatability and interobserver variability.^7^ Therefore, the principal weakness of our report is its dependence on expert opinion. While this might be a barrier to implementation, we hope that the points in this report might facilitate communication between histopathologists and physicians caring for patients. Furthermore, in the process we have identified a number of areas where the evidence base is weak and hope that this will stimulate further research.

It seems probable that, given the scattered nature of foci, the reliability of the test would improve in line with increasing surface area examined, particularly with a lower FS and with fewer ducts in the sample. However, an optimum surface area which balances FS reliability with practicality has yet to be determined. We have recommended obtaining four glands, although the minimum of 8 mm^2^ surface area may often be achieved with two to three glands. However, some glands may be atrophic or damaged and the material obtained should be sufficient to overcome these limitations and achieve a valid result. The surface area examined should be reported to aid the clinician in their interpretation and for transparency in clinical trial reports. A single study has demonstrated an increase in cutting levels to be useful for categorising patients with borderline FS,^35^ although, arguably, increasing the number of glands should be prioritised over the number of cutting levels. If multiple cutting levels are employed in a clinical trial setting, this should be protocolised, with scoring based on cumulative number of foci and glandular area across all slides, to avoid introduction of bias by selecting the ‘best’ slide. This latter consideration is less relevant for routine diagnosis, where an interpretation may be made based on the clearest level diagnostically, or a cumulative FS in case of insufficient surface area. We have suggested that additional cutting levels are done at 200 µm intervals, as this has been used in the referenced study, although further work would be required to define optimal intervals.

We have sought to clarify the issues we identified with the determination of FLS and FS calculation. For clinical trials we have also recommended a focus area calculation. One study found this correlated better with clinical and autoantibody parameters than the FS.^36^ Measurement of infiltrated area avoids difficulties in determining whether to count partially confluent foci as one or two, and remove the arbitrary ‘ceiling’ score in case of more widespread confluence. Furthermore, it is not unreasonable to expect that where foci are large, a significant reduction in area may occur following therapy despite the number of foci not being reduced, therefore, affording greater sensitivity.^6^ Foci are three-dimensional structures, and so the area of an individual focus will vary depending on the level at which it is sectioned. This issue could be minimised by either (i) ensuring sufficient glandular surface area is examined and (ii) taking more than one cutting level to avoid bias in the analysis.

There was a strong desire for further guidance on identification of GCs. Progressive organisation of foci into lymphoid-like structures is likely to have pathological consequences and function may occur without the fully formed appearance on H&E that can be seen in secondary lymphoid organs. In secondary lymphoid organs, areas of lighter staining often characterised by a rounded appearance are easily distinguished within the denser follicular area. Within these, a light and dark zone segregation is often also visible. The dark zone is the area of centroblast proliferation and the light zone is inhabited by centrocytes, T follicular helper cells and FDCs (whose large dendritic-like cytoplasm is responsible for the lighter staining with H&E). In LSGs, the detection of such structures is more challenging than in lymph nodes and GCs are often only appreciable with H&E as areas of lighter staining within the follicular area without the classical dark/light zone segregation. The varying prevalence (18%–59%)^37^ reported may reflect this difficulty and the consequent threshold for identification, alongside the differences in cohorts. This is important to clarify given the discordant data on the prognostic value of GCs for later lymphoma development.^3^ ^4^ ^38^ For the purpose of trials we have suggested additional immunostaining to study the organisation of foci. Other markers could be proposed, such as BCL-6 for GC detection,^26^ and may be appropriate depending on the study and agent proposed. Activation-induced cytidine deaminase (AID) is expressed if the GCs are functional but accurate staining for this is technically challenging.^39^

We have focused primarily on LSG tissue. The presence of LESA is more commonly observed in parotid tissue than in LSGs,^40^ with lymphoma development also occurring more often in the former. While this might be a consideration for the site of biopsy, the majority of centres still rely on LSGs due to the ease, familiarity and acceptance of this approach.

Extensive work on measurement of radiographic progression in rheumatoid arthritis (RA) has shown that inclusion of >1 reader reduces measurement error, and may allow smaller group sizes. Two readers is a good balance between accuracy and feasibility, and reader training is essential.^41^ We have extrapolated this to FS assessment in PSS. There has also been debate in the RA literature about whether sequential radiographs from the same patient should be scored together, and whether this should be done with knowledge of the chronological order.^41^ ^42^ As different glands are being sampled with LSG biopsy, this latter consideration is less relevant.

Further evaluation of alternative biomarkers to biopsy should be encouraged, including imaging modalities, salivary proteomics and peripheral blood immunophenotyping.^43–47^ Imaging would not provide biological proof of mechanism however, or mechanistic understanding in the context of a failed study.

In summary, we have provided a series of recommendations relating to the use of salivary gland histopathology in the diagnosis of PSS and in clinical trials, as a step towards the important objective of standardisation.
