# Supplementary material for: Standardisation of labial salivary gland histopathology in clinical trials in primary Sjögren's syndrome
Source: Ann Rheum Dis. 2017 Jun 9;76(7):1161–8. doi: 10.1136/annrheumdis-2016-210448 (PMC5530351; doi:10.1136/annrheumdis-2016-210448)
Supplement: supplementary table [file annrheumdis-2016-210448supp001.pdf]

Supplementary Table 1. Points relevant to clinical trials that were rejected in the eDephi process, showing strength of recommendation (A-D) based on available evidence, according to the scale (A to D) recommended by the Oxford Centre for Evidence-based Medicine.[22] The level of agreement (0-10 scale) amongst participants is also shown, represented by mean scores and the percentage of respondents who scored the point ≥6/10.

Point		Strength of recommendation	Number of respondents	Mean score (sd)	%≥6
**Guidance relevant to clinical trials**					
1	A focus score for follow-up samples should only be provided where the baseline sample has features consistent with FLS.	D	37	6.1 (3.4)	65
2	Consideration should be given to the measurement of the percentage of plasma cells positive for IgA and IgG	C/D	37	6.6 (2.3)	65
3	Consideration should be given to measuring glandular epithelial cell MHC class II expression	C/D	37	6.3 (2.3)	65
4	Despite the apparent stability or slow progression of histological features, a placebo group should preferably be included in a single agent study even in early phase mechanism-of-action studies where this is the primary outcome, until further experience with the heterogeneity of sampling is achieved.	D	38	7.0 (2.5)	74
